# Prevalence of lipid abnormalities before and after introduction of lipid modifying therapy among Swedish patients with dyslipidemia (PRIMULA)

**DOI:** 10.1186/1471-2458-10-737

**Published:** 2010-11-29

**Authors:** Billie Pettersson, Baishali Ambegaonkar, Vasilisa Sazonov, Mats Martinell, Jan Stålhammar, Per Wändell

**Affiliations:** 1 Center for Medical Technology Assessment, Linköping University, Linköping, Sweden; 2 Merck Sharp & Dohme (Sweden) AB, Sollentuna, Sweden; 3 Merck & Co., Inc., Whitehouse Station, New Jersey, USA; 4 Department of Public Health and Caring Sciences, Uppsala University, Uppsala, Sweden; 5 Centre for Family and Community Medicine, Karolinska Institute, Huddinge, Sweden

## Abstract

**Background:**

Data on the prevalence of dyslipidemia and attainment of goal/normal lipid levels in a Swedish population are scarce. The objective of this study is to estimate the prevalence of dyslipidemia and attainment of goal/normal lipid levels in patients treated with lipid modifying therapy (LMT).

**Methods:**

This longitudinal retrospective observational study covers time periods before and after treatment. Data were collected from 1994-2007 electronic patient records in public primary healthcare centers in Uppsala County, Sweden. Patients were included if they had been treated with LMT and had at least one lipid abnormality indicating dyslipidemia and if complete lipid profile data were available. Thresholds levels for lipids were defined as per Swedish guidelines.

**Results:**

Among 5,424 patients included, at baseline, the prevalence of dyslipidemia (≥1 lipid abnormality) was by definition 100%, while this figure was 82% at follow-up. At baseline, 60% had elevated low-density lipoprotein (LDL-C) combined with low high-density lipoprotein (HDL-C) and/or elevated triglycerides (TG s), corresponding figure at follow-up was 36%. Low HDL-C and/or elevated TGs at follow-up remained at 69% for patients with type 2 diabetes mellitus (T2DM), 50% among patients with coronary heart disease (CHD) and 66% among patients with 10 year CHD risk >20%. Of the total sample, 40% attained goal levels of LDL-C and 18% attained goal/normal levels on all three lipid parameters.

**Conclusions:**

Focusing therapy on LDL-C reduction allows 40% of patients to achieve LDL-C goal and helps reducing triglyceride levels. Almost 60% of patients experience persistent HDL-C and/or triglyceride abnormality independently of LDL-C levels and could be candidates for additional treatments.

## Background

Dyslipidemia is one of the major risk factors for cardiovascular disease (CVD) and coronary heart disease (CHD) [1,2]. Of all the deaths in 2005 in Sweden, 42% were caused by CVD[3]. The costs of CVD was estimated to around 10% of total health care expenditures in Sweden in 2006 [4].

Low-density lipoprotein cholesterol (LDL-C) is established as a key causative factor in the progression of CHD [5-7]. Low levels of high-density lipoprotein cholesterol (HDL-C) have been shown to be predictive and is an independent risk factor for developing CHD [8-10] At different levels of LDL-C, there is an inverse association between HDL-C and increased risk for CHD [7,11-13] and CVD [14].

At present guidelines focus on LDL-C levels [15] and statins are the gold standard for lowering LDL-C. However, type 2 diabetes mellitus (T2DM) and cardiovascular disease are associated with increased risk of metabolic syndrome, which includes dyslipidemia, [16] and the dyslipidemia in the metabolic syndrome is characterized by hypertriglyceridemia and low levels of HDL-C [17,18].

The aim of this study was to examine the prevalence of dyslipidemia, mixed dyslipidemia (MD), treatment patterns and attainment of goal/normal cholesterol levels before and after initiation of lipid modifying therapy (LMT).

## Methods

### Study design and data collection

This was a longitudinal retrospective observational study, which covers time periods before and after treatment. The study consisted of a baseline period, 15 months prior to initiation of a lipid modifying therapy (LMT) and a follow-up period (12-month following LMT). In clinical primary health care practice in Sweden, patients with chronic illnesses in stable state usually only visit their GP once every 12 months. By using a 15 months prescription free interval before index date one could assume that the included individuals were naive users of LMT.

Data were collected retrospectively (1994-2007) from electronic patient protocols using a search engine (The Pygargus Customized eXtraction Program (CXP) to scan patient protocols in 26 out of 30 public primary healthcare centers serving 77% of the total Uppsala county population (322 043 in 2007) in the county of Uppsala, Sweden.

Three CXP mediated extractions were performed in order to identify patient protocols: patients with International Classification of Diseases (ICD) diagnostic code of ICD-10 (-9) codes for dyslipidemia, prescription of a LMT (Anatomic Therapeutic Chemical [ATC]-code C10) and patients having existing laboratory results of total cholesterol (TC), TG, LDL-C and HDL-C measurement. The study was approved by the Regional Ethics committee in Uppsala in May 2008 (2008/120).

### Measures

The primary care data in which patients were identified included records of care-giver contacts, laboratory measurements, drug prescriptions, diagnoses and biometrics (blood pressure, height and weight) carried out within each centre. Lipid values captured were fasting or non-fasting. LDL-C measurement was considered invalid if the triglyceride value was >4.5 mmol/l.

### Patients

Patients >35 years of age were included if their lipid values indicated dyslipidemia and had initiated LMT (ATC code C10.x) between May 1994 and June 2006 and had complete lipid profiles at baseline and at follow-up. Treatment gaps of up to 6 weeks were allowed during the follow-up period except for the first 6 weeks post index date (initiation of LMT). A total of 5,424 patients met the criteria and were included in the study.

### Definitions

For the analyses, normal and goal lipid levels were defined according to Swedish guidelines: TC <4.5 mmol/l for patients at risk (< 5 mmol/l for non-high risk patients), LDL-C <2,5 mmol/l (< 3.0 mmol/l for non-risk patients), TG <1.7 mmol/l and HDL-C >1.0 and >1.3 mmol/l for men and women, respectively. Mixed dyslipidemia (MD) is defined as abnormal levels of more than one lipid fraction.

High-risk groups were defined as those with CHD, T2DM without CHD, and those with 10-year CHD risk >20% without CHD or T2DM. Patients with T2DM and those with CHD were identified from the ICD diagnostic codes. Patients with 10-year CHD risk >20% were identified by calculating risk per Framingham Risk Score (FRS) [19].

### Statistical analyses

Descriptive analyses were performed to evaluate baseline patient characteristics and the prevalence of dyslipidemia, MD and treatment patterns, using thresholds for dyslipidemia defined above. These analyses were carried out for the total study population as well as for subgroups. Chi-squared tests were carried out to detect significance in differences in proportions between groups at the level of P < 0.05 (two-tailed). Multivariate logistic regressions were used to evaluate factors associated to attainment of goal/normal lipid levels. The Statistical Package for Social Sciences (SPSS) versions 16 and 18 (SPSS Inc., Chicago, Illinois, USA)) were used for all analyses.

## Results

### Baseline characteristics

Mean age of the study population was 69 years and 46% were male (Table 1). About 30% were smokers, hypertension was present in 88% and obesity in 40%. Of the total study sample, about 40% were classified as high-risk patients. No major differences between men and women could be seen apart from 10-year CHD risk >20% which was more common among men than among women.

**Table 1:** Baseline characteristics, mean (SD) and percentage.

	Male (n = 2509)	Female (n = 2915)	Total (n = 5424)
Age (years)	68.1 (10.8)	70.5 (10.3)	69.4 (10.6)
**Cardiovascular (CV) risk factors**			
Current smoker	29%	31%	30%
Hypertension > 140/90 mmHg or medication	87%	89%	88%
Hypertension medication use at baseline	60%	62%	61%
Baseline systolic blood pressure (mmHg)	147.1 (17.4)	150.6 (18.6)	148.9 (18.1)
Baseline diastolic blood pressure (mmHg)	85.1 (9.0)	83.9 (8.6)	84.4 (8.8)
TC (mmol/l)	6.6 (1.3)	7.1 (1.2)	6.9 (1.3)
LDL-C (mmol/l)	4.3 (1.2)	4.7 (1.2)	4.5 (1.2)
TG (mmol/l)	2.5 (2.0)	2.2 (1.5)	2.4 (1.8)
HDL-C (mmol/l)	1.2 (0.3)	1.5 (0.5)	1.4 (0.4)
HbA1c (%-units)	6.2 (1.4)	6.0 (1.4)	6.1 (1.4)
Type 2 diabetes mellitus	28%	22%	25%
Age at diabetes onset (years)	59.7 (10.1)	61.3 (10.1)	60.5 (10.1)
Diabetes duration (years)	8.9 (3.5)	9.5 (3.5)	9.2 (3.5)
Obesity (BMI ≥ 30)	38%	42%	40%
CHD (1)	11%	8%	10%
Diabetes without CHD (2)	25%	21%	23%
Framingham Risk Score >20% without CHD or diabetes (3)	12%	2%	7%
Any of the criteria (1) - (3)	46%	30%	38%

To convert the values for cholesterol to mg/dL, multiply by 1/0.02586. To convert the values for triglycerides to mg/dL, multiply by 1/0.01129.

TC = total cholesterol, LDL-C = low-density lipoprotein, TG = triglycerides, HDL-C = high-density lipoprotein.

### Prevalence of dyslipidemia

#### Baseline

**Figure 1:**
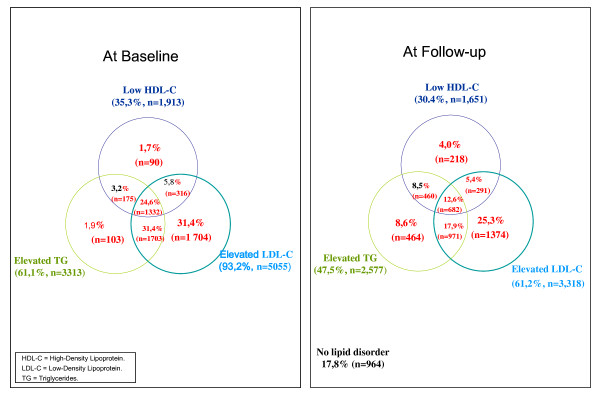
**Prevalence of lipid abnormalities before and after treatment with lipid modifying therapy in total population (n = 5,424)**.

**Figure 2:**
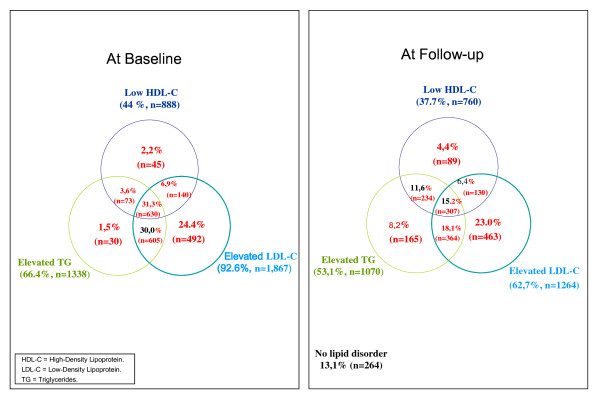
**Prevalence of lipid abnormalities before and after treatment with lipid modifying therapy in high-risk patients (n = 2,016)**.

Elevated TC as well as elevated LDL-C were observed in over 90% and elevated TG were found in 61% and 66% of the total population and all high-risk patients respectively (Table 2). Isolated elevated LDL-C was less prevalent in high risk population compared to total population, 24% compared to 31% (Figure 1 and 2). Low HDL-C and/or elevated TG were detected in almost 70% of the total population and 76% of all high-risk patients (Table 2). Low HDL-C, elevated TGs as well as MD were most predominant in patients with T2DM and in patients with 10-year risk for CHD >20% (Table 3).

**Table 2:** Prevalence of dyslipidemia at baseline and follow-up in total and all high risk populations

	Total (n = 5424)		All high risk (n = 2016)	
	**Baseline**	**Follow up**	**Baseline**	**Follow up**
**Elevated TC**	95%	68%	93%	67%
**Elevated LDL-C**	93%	61%	93%	63%
**Elevated TGs**	61%	48%	66%	53%
**Low HDL-C**	35%	30%	44%	38%
**Low HDL and/or elevated TGs**	69%	57%	76%	64%
**Elevated LDL-C and low HDL-C and/or elevated TGs**	62%	36%	68%	40%
**Elevated LDL-C and/or low HDL-C and/or elevated TGs**	100%	82%	100%	87%

TC = total cholesterol, LDL-C = low-density lipoprotein, TG = triglycerides, HDL-C = high-density lipoprotein.

**Table 3:** Prevalence of dyslipidemia at baseline (BL) and follow-up (FU) by riskgroups and p-values for comparison of proportions.

	T2DM (n = 1238)		CHD (n = 520)		10-year CHD risk > 20% (n = 258)		Difference (T2DM vs CHD) P-value		Difference (T2DM vs 10-year CHD risk > 20%) P-value		Difference (CHD vs 10-year CHD risk >20%) P-value	
	**BL**	**FU**	**BL**	**FU**	**BL**	**FU**	**BL**	**FU**	**BL**	**FU**	**BL**	**FU**
**Elevated TC**	95%	67%	90%	66%	93%	69%	< 0.001	0.691	0.39	0.381	0.098	0.289
**Elevated LDL-C**	92%	60%	92%	65%	95%	72%	0.834	0.048	0.221	< 0.0001	0.208	0.032
**Elevated TGs**	72%	59%	49%	39%	74%	53%	< 0.0001	< 0.0001	0.585	0.054	< 0.0001	< 0.0001
**Low HDL-C**	47%	42%	35%	27%	51%	36%	< 0.0001	< 0.0001	0.223	0.043	< 0.0001	0.017
**Low HDL and/or elevated TG's**	80%	69%	60%	50%	83%	66%	< 0.0001	< 0.0001	0.435	0.294	< 0.0001	< 0.0001
**Elevated LDL-C and low HDL-C and/or elevated TG's**	73%	41%	53%	32%	77%	48%	< 0.0001	< 0.0001	0.157	0.045	< 0.0001	< 0.0001
**Elevated LDL-C and/or low HDL-C and/or elevated TG's**	100%	88%	100%	83%	100%	90%	0.123	0.13	-	0.242	0.481	0.008

TC = total cholesterol, LDL-C = low-density lipoprotein, TG = triglycerides, HDL-C = high-density lipoprotein.

#### Follow-up

At follow-up, dyslipidemia in at least one lipid fraction was seen in 82% and 87% of the total and high-risk populations respectively. Elevated TGs persisted in roughly half of all patients, and low HDL-C was found in about 30% of all patients and 38% of the high risk patients. Low HDL-C and/or elevated TG persisted in 57% of the total population and 64% of the high-risk patients, while elevated LDL-C combined with low HDL-C and/or elevated TG was found in 36% of the total population, and in 40% of the high-risk population, respectively (Table 2). Isolated elevated LDL-C was less prevalent in high risk population (23%) compared to total population (25%) at follow-up (Figure 1 and 2).

### Differences between high risk groups

At baseline, there were large differences between different high risk groups in terms of prevalence of elevated TGs, low HDL-C and MD while no major differences were seen concerning elevated TC or LDL-C. The most prominent differences were in the prevalence of dyslipidemia in the group with T2DM compared to those with CHD. No major differences were observed between patients with T2DM and those with 10 year CHD risk >20% (Table 3).

At follow-up, differences in TC and LDL-C were prominent in the group with 10-year CHD risk >20%, where it persisted in about 70%. The prevalence of elevated TGs, low HDL-C and combinations decreased modestly and persisted to a greater extent in the groups with T2DM and with 10-year CHD risk >20% (Table 3).

### Attainment of goal/normal lipid levels

In the total study population TC and LDL-C goals were attained by about 30% and 40%, respectively, and normal levels in TGs and HDL-C were attained by about 50% and 70% respectively. Only 18% of the total population and 13% of the high-risk population attained goal/normal levels for all three lipid parameters (Figure 1 and 2).

Multivariate regression models were run to determine attainment of goal/normal levels in TC, LDL-C, TGs and HDL-C. The basic model included following covariates: age and gender, BMI, smoking, hypertension, baseline lipid levels, history of diabetes, history of CHD, and time on statin therapy. Non-significant variables in each model were excluded in a stepwise manner, retaining only age, gender and time on statin (even if non-significant) in the final models.

Results (Table 4) suggested a slight, positive association between age and attainment of goal/normal levels in all lipid parameters. Female compared to male gender was associated with significantly lower odds of attaining normal levels in HDL-C (Odds Ratio [OR], 0.07; 95% CI, 0.05-0.08).

**Table 4:** Logistic regressions on goal/normal lipid level attainment

Dependent variable		Odds ratio	95% CI	
***TC***	Age	1.02	1.01	1.03
	Gender	0.89	0.78	1.01
	T2DM	0.73	0.63	0.85
	CHD	0.55	0.45	0.69
	TC	0.93	0.92	0.93
	Time on statin	0.85	0.83	0.87
				
***LDL-C***	Age	1.02	1.01	1.02
	Gender	1.20	1.06	1.36
	T2DM	0.65	0.56	0.75
	CHD	0.42	0.34	0.53
	LDL-C	0.93	0.92	0.94
	Time on statin	0.86	0.84	0.88
				
***TGs***	Age	1.01	1.01	1.02
	Gender	1.05	0.92	1.20
	T2DM	0.73	0.63	0.86
	TGs	0.84	0.84	0.85
	Time on statin	0.94	0.92	0.97
				
***HDL-C***	Age	1.01	1.00	1.02
	Gender	0.07	0.05	0.08
	T2DM	0.70	0.58	0.83
	HDL-C	2.15	2.06	2.25
	Time on statin	1.02	0.99	1.05

TC = total cholesterol, LDL-C = low-density lipoprotein, TG = triglycerides, HDL-C = high-density lipoprotein. The basic model included age and gender, BMI. Smoking, hypertension, baseline lipid values, history of diabetes, history of CHD and time on statin therapy (years). Insignificant variables were then stepwise excluded. Age, gender and time on statin are considered important to keep in model even if non significant in some models. Gender: Male = 1 (Female have higher odds to attaining LDL-C goals and lower odds for attaining goals of HDL-C compared to males). Risk factors: T2DM = 1, no T2DM = 0, Having CHD = 1, No CHD = 0 (Example: having T2DM is associated with lower odds of attaining goal in any lipid parameter and having CHD is associated with lower odds of attaining goals in TC and LDL-C).

Patients with T2DM had significantly lower odds of attaining lipid goals/normal levels in any lipid parameter than patients without T2DM. Patients with a history of CHD had significantly lower odds of reaching goal/normal level in TC or LDL-C, than patients without history of CHD.

Baseline lipid values were strongly associated with attainment of goal/normal levels in all lipid parameters. For each 0.1 mmol/l increase in TC or LDL-C at baseline the odds of attaining goal levels decreased about 7% (OR, 0.93; 95% CI, 0.92-0.93) and (OR, 0.93; 95% CI, 0.92-0.94) for TC and for LDL-C respectively. For each 0.1 mmol/l increase in TG at baseline the odds of attaining normal levels was about 15% lower. Baseline HDL-C values were strongly and positively associated to attainment of normal levels as the odds increased by more than 200% for each 0.1 mmol/l increase in baseline value (OR, 2.15; 95% CI, 2.06-2.25).

Duration of statin therapy was associated to lower odds of attaining goal/normal levels of TC, LDL-C and TG. For each year on statin treatment the odds of attaining TC or LDL-C goal were about 15% lower and the odds of attaining normal levels of TGs was 6% lower.

### Dyslipidemia pharmacotherapy

Statin monotherapy was the dominating LMT with small variations between different subgroups (Table 5). Fibrates were mostly used among patients with T2DM patients. The use of other therapies was equally limited.

**Table 5:** Lipid modifying therapy by total and all high risk population and respective high risk group.

	Total (n = 5361)	All high risk (n = 2013)	T2DM (n = 1237)	CHD (n = 518)	10 year CHD risk > 20% (n = 258)
**Statins alone**	93.6%	93.8%	92.5%	96.3%	95.0%
**Fibrates alone**	5.3%	5.8%	7.1%	2.9%	5.0%
**Other monotherapy**	0.9%	0.2%	0.2%	0.4%	0.0%
**Combinations and other**	0.3%	0.2%	0.2%	0.4%	0.0%

## Discussion

### Main findings

Among Swedish patients, most of whom were treated with statins only, prevalence of dyslipidemia decreased significantly following treatment, from 95% to 68% in patients with elevated TC and from 93% to 61% for patients with elevated LDL-C. Isolated elevated LDL-C was less prevalent in high risk population. Elevated TGs persisted in about 50% and low HDL-C persisted in about 30% at follow up for total sample, while MD defined as prevalence of low HDL-C and/or elevated TGs were prevalent in 60%.

Improvement in TGs was moderate and low HDL-C persisted, showing only modest improvement following therapy, and this was most notable in patients with T2DM. Compared with nondiabetic patients, those with diabetes in our study had a greater risk of experiencing dyslipidemia involving TGs and HDL-C. This finding is supported by observations of increased TGs and low HDL-C in other studies involving patients with diabetes [20,21].

About 30% and 40% of all patients attained goals of TC and of LDL-C respectively following treatment, while only additional 5% and 10% patients attained HDL-C and TG normal levels respectively following treatment. Attaining lipid goal/normal levels was slightly associated to age while not significantly associated to gender except for attainment of normal levels in HDL-C. History of CHD was associated with lower odds for attainment of TC and LDL-C goals. Diagnosis of T2DM was negatively associated to attainment of goal/normal levels for any lipid parameter. Baseline lipid levels of TC, LDL-C and TGs were strongly and negatively associated to attainment of goal/normal levels in TC, LDL-C and TGs, while baseline HDL-C was positively associated to attainment of normal levels in HDL-C. Duration of statin therapy was negatively associated to attainment of goal/normal lipid levels.

### Findings compared to other studies

A large cohort study in northern Sweden [22] showed that mean lipid levels (TC) have decreased significantly during 1986-2004 from 6.4 to 5.8 mmol/l in men and 6.3 to 5.5 mmol/l in women in age group 25-64 years and from 6.4 to 5.5 mmol/l in men and from 7.1 to 6.2 mmol/l in women in higher age group [3]. In this study mean TC was higher for men (6.6 mmol/l) and women (7.1 mmol/l), indicating a difference of about 1.0 mmol/l compared to the findings in the above study. This could be explained by the inclusion criteria in this study where patients were selected if they were treated with LMT and had indication of elevated lipid levels. In a study using data up to 2003 on patients treated with a LMT in the same county (Uppsala Sweden) as in this study, mean TC was 7.47 mmol/l in the total population, which could be compared to 6.9 mmol/l in this study[23], indicating a decrease in TC lipid levels from 2003-2007. Another cohort study [24] in a Swedish population showed that as from 2004 there is a tendency to an increase in mean TC lipid levels among both men and women [3]. This tendency could however be an indication for regional differences in treatment patterns, demography and socio economic factors. High cholesterol concentrations are associated to low socio economic status [25].

A study from France [26], where fibrates are more frequently used found that regardless of LDL-C levels 38.7% had MD defined as elevated TGs and/or low HDL-C, which corresponds to 57% in this study. Fibrates have been shown effective in raising HDL-C and lowering TGs [27] and this could explain some of the difference in findings. Another important difference could be explained by baseline lipid profiles. Baseline mean lipid values were lower (higher in HDL-C) 3.3, 1.46 and 1.59 mmol/l in LDL-C, HDL-C and TGs respectively in the French study, which could be compared to 4.5, 1.4, 2.4 mmol/l in this study. Lipid levels at baseline were found to be strongly associated to attainment of goal/normal lipid levels following treatment. This finding is in line with another study where high lipid levels at baseline were found to be strong predictors of failing to reach goal/normal levels in TC, LDL-C and TGs and the inverse for the HDL-C [28].

A study on Swedish patients treated with statins found that 70% of high-risk patients had at least one lipid abnormality [29] compared to 82% in this study, a difference that could be due to different treatment patterns at GP and specialist settings, since patients in that study were recruited by specialists (40%) whereas in this study patients were recruited in GP settings only.

In an earlier study[30], where goal attainment was defined as having a TC below 5.0 mmol/l and LDL-C level below 3.0 mmol/l (as recommended by the Swedish Medical Products Agency at that time) 31% reached goal levels, compared to 43% in this study, which could indicate improved treatment of TC or LDL-C disorders between study periods. In the earlier study the time period was 1993-2001 and in our study the period is 1994-2007, a time period where acceptance and use of statins was widely increased in Sweden. In 2003 the patent of simvastatin expired and the price for the generic simvastatin decreased to about 10% of the price of the patent drug, which could partly explain some of the significant increase in usage of statins.

A study with a similar design and method carried out in the US and included 5,158 patients found that therapy (primarily statins) reduced the proportion of patients not at LDL-C goals from 77% to 22% and the proportion with high TG levels from 34% to 20%. HDL cholesterol levels were unchanged (49% and 50% were less than normal levels before and after therapy respectively) in the aggregate and in high-risk subgroups (patients with coronary artery disease, diabetes, and 10-year heart disease risk >20%). After therapy 29% of high-risk patients were found having multiple lipid abnormalities [31]. These findings are consistent to findings in our study, however proportions of patients not reaching LDL-C goal were higher in our study (93% to 61% before and after treatment). This difference could partly be due to different thresholds for dyslipidemia employed in the studies.

We also carried out analyses using the thresholds of target/normal levels for LDL-C, HDL-C and TG specified as per National Cholesterol Education Program, Adult Treatment Panel III (NCEP ATP III) guidelines, LDL-C < 100 (2.59 mmol/l), 130 (3.37 mmol/l) or 160 mg/dL (4.14 mmol/l) (depending on risk factors), HDL-C > 40 mg/dL (1.04 mmol/l) in men, > 50 mg/dL (1.30 mmol/l) in women and Triglycerides < 150 mg/dL (1.69 mmol/l), but not considered high until > 200 mg/dL (2.26 mmol/l) [32,33]. The NCEP guidelines are slightly different from the Swedish guidelines, threshold for abnormal LDL-C is somewhat higher in the NCEP compared to Swedish guidelines (2.59 mmol/l vs to 2.5 mmol/l). Using NCEP thresholds elevated LDL-C was prevalent in less than 40% of total study population, whilst this figure was over 50% in the high risk population. These differences were the major differences compared to the results using Swedish guidelines. Using NCEP thresholds yields more consistent findings as compared to the study carried out in the US and was discussed above [31].

### Treatment patterns and importance of addressing lipid abnormalities

Statin therapy is established and accepted as primary and secondary prevention [15] and the effect of statins on lowering LDL-C as well as their effect on reducing the risk of cardiovascular events has been widely documented [5,34-36]. Since in this study, treatment was primarily targeted towards lowering LDL-C, isolated LDL-C was less prevalent in high risk population. Low HDL-C and elevated TGs are independent risk factors for CHD [8,12,32,37] and abnormalities in these lipids were not much improved in this study: Elevated TGs and low HDL-C and combinations persisted to a large extent, mostly in high-risk population despite LMT.

In the logistic regression analysis we found duration of statin therapy to be negatively associated to attainment of goal/normal levels. For each year increase on statin treatment the odds of attaining LDL-C goal decreased by about 10% (OR, 0,86; 95%CI, 0,84-0,87). This could indicate lower efficacy with increasing time on treatment, however better medication possession ratio was found to be associated with a better goal attainment in TC and in LDL-C [28]. Half of all patients on statin treatment discontinue the medication by the end of the first year [38]. Our findings might be influenced by factors related to patient compliance to treatment and discontinuation. Patients in this study were assumed to be compliant to treatment, as they fulfilled the criteria of refilling their prescriptions for at least one year, however this might still not accurately reflect real compliance to treatment. Another possible explanation could be related to dosing. Almost 94% of all patients in this study were treated with statins, of which 61% were treated with simvastatin. The mean dose for those patients treated with simvastatin was 16.74 mg, 43% were treated with 10 mg/day, 52% with 20 mg/day, 4.9% with 40 mg/day and only 0,1% were treated with 80 mg/day. This dosing is in the lower range of what is recommended for patients at high risk [15]. The doses of other statins were also in the lower ranges.

Therapies that modify LDL-C, HDL and/or TG exist but they are not widely utilized. Use of new medical technology varies widely between countries but also between different disease areas within a country [39]. The causes for variations in use of new drugs could be divided into three broad groups, macro-or system level determinants, service organization determinants and clinical practice determinants [40]. Examples of determinants could be spending on pharmaceuticals, role and impact of health technology assessment, guidelines and clinical culture and attitudes etc. Use of statins as well as use of drugs in other therapy areas was low in Sweden when compared to other countries [39], which could be an indicator and explanation for low uptake of new drugs in general.

Some of the therapies that modify LDL-C, HDL and/or TG are associated with limitations and might explain the low utilization of these therapies. Fibric acid and nicotinic derivatives are two classes with documented effects on increasing HDL-C and association to reduced CV events, but have limited documentation on CV outcomes and are associated with high rates of adverse events or tolerability issues [27]. The most common side effects during fibrate therapy were skin reactions and gastrointestinal symptoms, while flushing was the most common issue during therapy on Niacin [27] and could be major reason behind the limited use of these treatment options.

### Limitations and strengths

Total patients that met all predefined inclusion and exclusion criteria and were included in the analysis were 5424, about 58% of total study population (n = 5424/9384). The inclusion criteria of complete lipid profiles caused an exclusion of about 21% (n = 1933/9384), which may result in selection bias, since patients with high CVD risk should have better documentation and a greater probability of selection in the cohort and this could be a limitation of generalizability. The problem of missing complete lipid profiles was reported by other researchers and the analytical solution used in this study was adapted from their prior work [28]. In short, they compared baseline lipid values for those patients with complete lipid profiles compared to the included patients. If baseline lipid profiles were similar they concluded that those patients with complete profiles were representative for the entire cohort. Using the same methodology in this study no major differences in baseline lipid values were found between excluded and included cases. Nonetheless, one should use caution when extrapolating these data to the general population of patients using LMT.

Another limitation is that our analyses were on patients who got their LMT prescription refilled for at least one year and had complete lipid profiles, which might thus represent a best-case scenario of goal/normal lipid level attainment. Another possible limitation is that it was not possible to differentiate between fasting and non-fasting TG measurements in this study. To address this issue LDL-C measurements were considered invalid if the triglyceride values were >4.5 mmol/L. Furthermore both fasting as well as non-fasting TG act as strong predictors of cardiovascular events [41].

We calculated 10-year CHD risk >20% using FRS, since this predicts fatal and non fatal CHD events, while the European risk classifying system, SCORE[42], only predicts fatal events. The FRS system is widely used and validated [19]. However, for certain populations the FRS has been shown to overestimate risk [43-45]. In this study only 258 of all patients (not included in patients with T2DM or CHD) in total sample were found to be at 10-year CHD risk >20%.

The major strength of this study is that patients were identified from real clinical practice, which provides insight on prevalence, treatment patterns and goal attainment in patients with dyslipidema in Sweden and could be of use for decision making within the health care system. For instance, all lipid modifying agents on the Swedish market were reviewed in 2009 by the Swedish decision making body on reimbursement of pharmaceuticals (The Dental and Pharmaceutical Benefits Agency, TLV). The outcomes of the review acknowledged the need for drugs other than statins that could be useful for patients as add on to a statin therapy where statin monotherapy is not sufficient or when statins are not tolerated [46], which is an important stand point within health policy and may help reducing the burden of CVD.

Complete lipid profiles provide possibilities to assess different types of disorders and can be used to identify patients where additional treatment is needed to target specific dyslipidemia profiles. Our findings indicate this need in particular for patients with T2DM. In the "The Action to Control Cardiovascular Risk in Diabetes" (ACCORD) study however, combination treatment of simvastatin and fenofibrate in patients with T2DM was not found to reduce the rate of fatal cardiovascular events, nonfatal myocardial infarction, or nonfatal stroke, as compared with simvastatin alone [47]. However, the patients in the ACCORD study were at very high risk for CVD, therefore findings can not be generalized to all patients with T2DM. Furthermore there are strong evidence for low HDL-C as well as elevated TGs [8,12,32,37] besides elevated TC and LDL-C as risk factors for CVD and patients at risk should be considered for treatments in addition to statins that target multiple lipid disorders.

## Conclusions

Focusing dyslipidemia therapy on LDL-C reduction allows 40% of all patients to successfully achieve LDL-C goal and also helps reduce triglyceride levels, whereas HDL-C and/or triglyceride abnormalities mainly persist. Despite treatment with statins high-risk patients are still at substantial risk of CVD events. Low HDL-C is a known risk factor for CHD but appears to be largely ignored despite existence of agents that raise HDL-C. About 60% of all patients starting statin therapy could be considered for addition of treatments that target multiple lipid disorders. This option is most urgent for patients with type 2 diabetes mellitus.

## Competing interests

Conflicts of interest: Billie Pettersson, Baishali Ambegaonkar and Vasilisa Sazonov are employees at Merck &Co. Inc. and may own stock or have stock options in the company.

## Authors' contributions

BP coordinated the study, drafted the manuscript and performed the statistical analyses. BA and VS conceived of the study, participated in the design of the study and helped to draft the manuscript. MM and JS helped with medical expertise and data collection methods and PW helped to draft the manuscript and provided assistance on the statistical analyses and data presentation. All authors read and approved the final manuscript.

## Pre-publication history

The pre-publication history for this paper can be accessed here:

http://www.biomedcentral.com/1471-2458/10/737/prepub
